# Putting the affect into affective polarisation

**DOI:** 10.1080/02699931.2024.2362366

**Published:** 2024-06-07

**Authors:** Bert N. Bakker, Yphtach Lelkes

**Affiliations:** a Amsterdam School of Communication Research, University of Amsterdam, Amsterdam, the Netherlands; b Annenberg School for Communication, University of Pennsylvania, Philadelphia, CA, USA

**Keywords:** Affective polarisation, core affect, physiology, discrete emotions

## Abstract

While many believe that affective polarisation poses a significant threat to democratic stability, the definition and operationalisation of the concept varies greatly. This leads to conceptual slippage as well as imprecise tests of the causes and consequences of affective polarisation. In order to clearly identify and target its micro-foundations, we must understand the degree to which political divides are, in fact, affective. In this paper, we do so. We begin by delineating affective polarisation, a social divide that is purportedly distinct from policy-based disagreements. Subsequently, we explore the influence of emotions in politics, including how affect is conceptualised within the framework of polarisation. Where possible, our literature review is supplemented with analyses of existing datasets to support our points. The paper concludes by proposing a series of questions emotion researchers could address in the study of polarisation.

Affective polarisation, we are frequently told by politicians, pundits, journalists, and many of our colleagues, is a major threat to democratic stability. This concept, which suggests that we should look at polarisation through the lens of social identity, borrows heavily from psychological research (e.g. Huddy et al., 2015; Mason, 2024).

However, researchers have largely ignored the emotions literature central to psychology. Consequently, researchers have mostly focused on one dimension of affect using one type of measurement.

While political scientists have made some headway in understanding the causes and consequences of this phenomenon (or at least what is picked up by existing measures; Iyengar et al., 2019), the concept of affective polarisation has yet to be explicated. In particular, affective polarisation researchers do not have an agreed-upon definition and operationalisation of affect (Rollicke, 2023). This has led to conceptual slippage (Sartori, 1991); for instance, people use terms like negative partisanship, pernicious polarisation, affective polarisation, and social polarisation interchangeably (Lelkes, 2021b). Clear conceptualisation is necessary to understand and solve this societal problem. Understanding if and how polarisation can be affective allows us to clearly identify and target its microfoundations.

This article both introduces emotions researchers to the extant literature on affective polarisation and invite psychologists to lend their expertise to this area of research. To achieve this goal, we first offer an overview of existing (affective) polarisation research and the application of social identity theory (Tajfel, 1974) to the study of partisanship (e.g. Huddy, 2001; Huddy et al., 2015; Mason, 2024). This is followed by a discussion on the role of emotions in politics, generally, and a discussion about the conceptualisation of affect in polarisation. We end this paper with a series of questions emotion researchers could, potentially, address in the study of polarisation.

## Conceptualising mass polarisation

Confusingly, the term “polarisation” has been used in many ways over the years, and researchers have contested the meaning and importance of each use as well as the extent to which groups are polarised under that definition (for a review, see Lelkes, 2016). Political scientists distinguish between elite polarisation (e.g. politicians and parties) and mass polarisation (e.g. citizens and partisan identifiers). They also operationalise polarisation in many different ways. For instance, scholars have examined spatial polarisation, meaning the clustering of like-minded individuals, voting polarisation, meaning the percentage of Democrats/Republicans who vote for the Democratic/Republican candidate, and other measures (e.g. Klar et al., 2018; Mason, 2015, 2018). The most commonly discussed definition of polarisation is “ideological polarisation”. By ideological, scholars meant citizens’ positions on specific policy issues (such as abortion, immigration, economic policies, etc.).

Although scholars often use the term “polarisation” at the individual-level as a measure of extremity on some attitude (John is polarised), polarisation is most properly understood as a description of the distribution of a population (America is polarised). In particular, the degree to which a population is polarised is defined as the degree to which the distribution of some measure approximates a bimodal distribution. Polarisation describes both a state (the degree to which the distribution is bimodal at any one time period) and a trend (the degree to which the distribution is moving towards bimodality). Nonetheless, we adopt the language of existing research and use the term to describe individual-level attitudes.

Regardless of the type of polarisation under investigation, polarisation ultimately refers to a collapse of dimensionality and the flattening of conflict. For instance, attitudes around the world have long varied on two dimensions–economics and culture–and the two dimensions are only weakly correlated (Malka et al., 2019). In the American context, policy attitudes were once only weakly correlated with partisanship, as there were both conservative Republicans and Democrats and liberal Republicans and Democrats. Today, policy attitudes can increasingly be explained by a single ideological dimension (Hare, 2022), particularly among political elites. That is, if you tell us your position on abortion, we can guess your position on immigration.

In the early aughts, a heated discussion arose in political science about the extent to which Americans were, in fact, ideologically polarised (e.g. Abramowitz & Saunders, 2005; Fiorina & Levendusky, 2006). On one side of the debate, scholars examined how raw attitudes approached bimodality. That is, were Americans moving towards the extremes of the distribution? Studies have consistently shown that this is not the case: at the level of the mass public, the standard deviations of these distributions have remained relatively stable (Hill & Tausanovitch, 2015). On the other side of the debate, scholars have examined the degree to which Americans increasingly hold party-consistent attitudes. Do Democrats/Republicans hold liberal/conservative attitudes across issues? This form of polarisation, also called partisan sorting, is clearly happening. Although some scholars insist that partisan sorting is not polarisation, American policy attitudes and vote choices can be increasingly explained by a person's partisan identity. Nonetheless, the search for ideological polarisation in America always stood on an unstable footing. Americans are, famously, ideologically innocent (Converse, 1964). That is, attitudes are unstable and malleable, and most Americans do not have a firm grasp on words like “liberal” or “conservative”.

**Figure 1.**
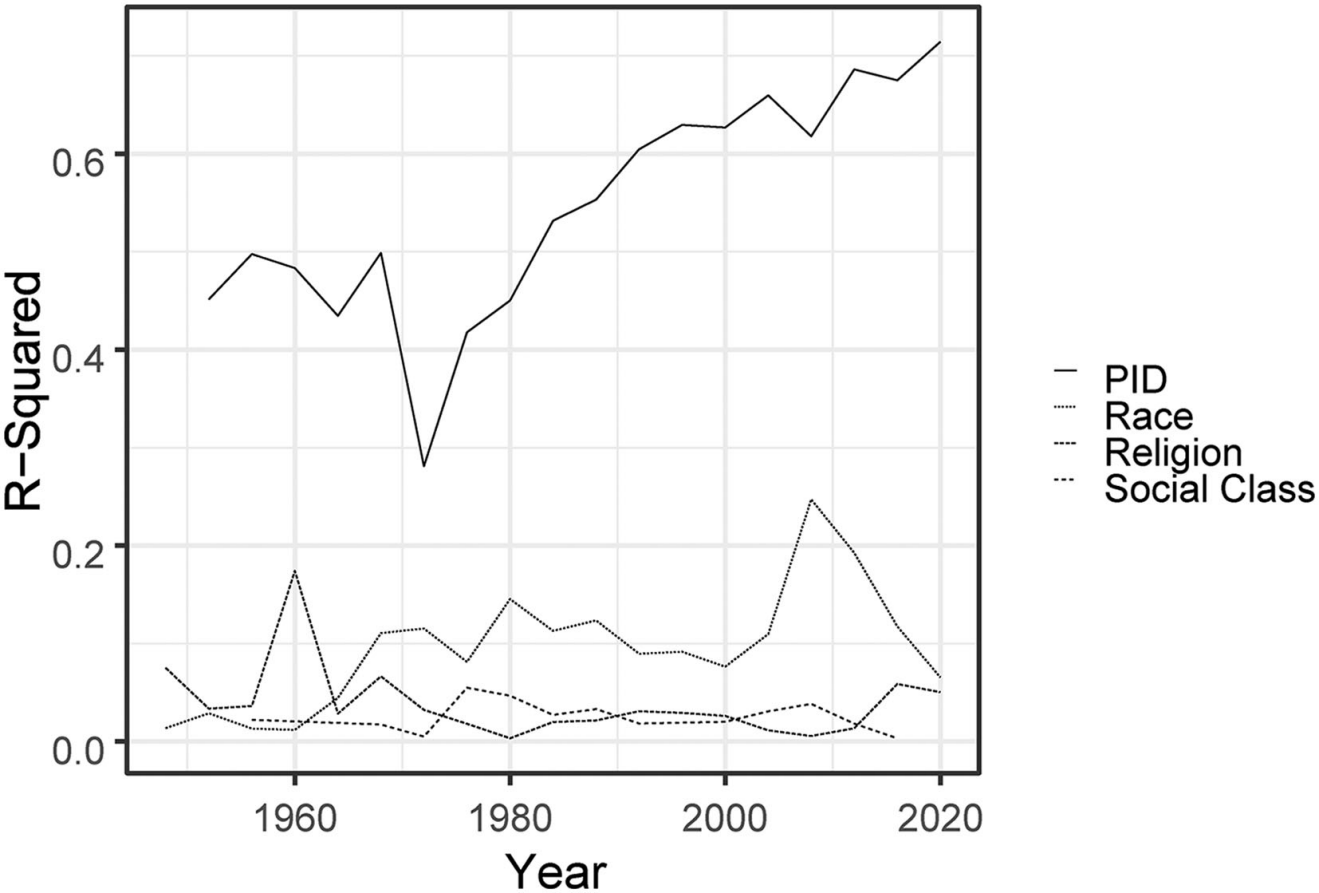
The variance of vote choice explained by Partisan identity (PID), race, religion and social class.

While the debate on the question whether polarisation equates to the increasing correlation between ideology and partisanship raged on, scholars began discussing a form of polarisation not necessarily based on policy attitudes but grounded in a person's partisanship or partisan identity. Partisanship is, increasingly, the central cleavage in American politics. Take, for instance, data from the American National Election Study, a long-running survey of American attitudes. In a series of bivariate linear probability models, we regressed two-party vote choice (whether a person voted for the Democratic or Republican presidential candidate) on a person's self-reported race, religion, class, and party identity and extracted the r-squared for each variable-year. The results are plotted in Figure 1.

There are multiple noteworthy trends. First, in American politics, partisan identity has always explained more of the variance in a person's vote choice than the other salient identities. For instance, race, the second most important identity of the four, has, at the height of its importance in 2008, explained 25 percent of the variance in a person's vote choice. Partisan identity, when it was the least consequential in 1972, still explained 28 percent of the variance in a person's vote choice – which is still a lot more than the explained variance that race, religion or social class explained in 1972. Second, while the importance of other identities has remained relatively flat over the past 60 years, partisanship has only become increasingly important – as is signalled by the increasing line for partisan identity over time. By 2020, party identity explained 71 percent of the variance in vote choice.

## Social identity theory and the study of polarisation

Partisanship is central to American politics, but what is partisanship? Political scientists have defined partisanship in two ways. Under the instrumental perspective, “partisanship is a running tally of party performance, ideological beliefs, and proximity to the party in terms of one's preferred policies that is affected by current features of the political environment” (Huddy et al., 2015, p. 1). If partisanship is, in fact, instrumental, focusing on ideological polarisation makes some sense, as party is a proxy for policy positions.

However, the dominant model of partisanship in America situates it as a social identity (Huddy et al., 2015). Party identification is defined as “the individual's affective orientation to an important group-object in his environment” (Campbell, 1980, p. 121). Political scientists have marshalled evidence for this claim in several ways. Like other identities, partisan attachments are quite stable over time (Green et al., 2004). Individuals who align themselves with a particular political party also adapt their actions and beliefs to better align with the values of their chosen group. For instance, in one field experiment (Gerber et al., 2010), citizens received mailings informing them that they needed to register with a party to participate in an upcoming presidential primary. Those induced to identify with a party became more similar to other partisans in their voting behaviour and political attitudes. Similarly, researchers used Trump's inconsistent attitude positions to test the notion that people follow prototypical group leaders (Barber & Pope, 2019). When given information that Trump supported or opposed a policy, partisan identifiers, particularly strong identifiers, shifted their attitudes toward his stated position. This kind of normative social influence is a key concept in social identity theory. Of course, partisanship may have different foundations for different people (Arceneaux & Vander Wielen, 2017; Bakker et al., 2015; Bankert, 2022; Bowes et al., 2020; Satherley et al., 2020).

Because partisanship may be a social identity, a social identity framework rather than a policy framework may be a more appropriate lens for understanding political division.^1^ A social identity model of polarisation, rather than a policy-based model of polarisation, has different implications regarding how people express themselves and how we measure polarisation. In particular, “self-categorization determines emotional reactions, and identification with the group, by and large, heightens its impact” (Mackie et al., 2008, p. 1870). More concretely, when people are divided into even minimal groups, they develop warm feelings towards members of their own group and, sometimes, develop negative feelings towards members of opposing groups (Tajfel, 1974). Hence, polarisation may not be ideological, but it may be “affective”. Scholars have now documented how, compared to the past, partisans rate partisans of the out-group (e.g. Democrats rating Republicans and Republicans rating Democrats) far more negatively on various survey instruments Iyengar et al. (2012).

While the affective polarisation literature has primarily focused on American politics, there is a robust literature that also considers the presence, causes and consequences of (rising) affective polarisation outside the United States (e.g. Bassan-Nygate & Weiss, 2022; Bäck et al., 2023; Boxell et al., 2024; Garrett & Bankert, 2020; Garzia et al., 2023; Gidron et al., 2020; Harteveld, 2021; Hobolt et al., 2021; Hobolt et al., 2023; Janssen, 2024; Kekkonen & Ylä-Anttila, 2021; Orhan, 2022; Renström et al., 2021; Segovia, 2022; Turner-Zwinkels et al., 2023; Wagner, 2021). The concept of partisanship is far more complicated outside of the two-party U.S., and, in a multiparty system, requires unique assumptions about which parties a person identifies with and which parties a person does not (Reiljan, 2020; Wagner, 2021). Not only are there many more parties that need to be somehow weighted according to their electoral strength, but parties, particularly in countries with proportional representation, form coalitions, and voters tend to exhibit warmer feelings towards parties that are in the coalition (but with whom they do not explicitly identify with) (Gidron et al., 2023). Using measures of partisan social identity, Bankert et al. (2017) find there is considerable variation in partisanship in multiparty systems.

## The role of emotions in affective polarisation

While the early literature on affective polarisation did not substantively engage with psychological theories of emotions (e.g. Iyengar et al., 2012; Iyengar & Westwood, 2015), political scientists have long recognised that politics is affective (for a review, see Webster & Albertson, 2022). The “hot cognition” hypothesis (Abelson, 1963), for instance, suggests that socio-political concepts are affectively charged in the brain.

According to this model, parties, leaders, and issues are affectively tagged as either positive or negative and stored in long-term memory. When an individual retrieves the information about a party (or a leader or an issue), this tag is activated. Evidence for this so-called “hot cognition” hypothesis is reported in various studies: Bakker, Schumacher, et al. (2021) show that watching political messages increases affective responses as captured with skin conductance level increases compared to baseline; Electroencephalography (EEG) studies – EEG is a non-invasive neuroimaging technique that can measure and record the electrical activity of the brain – find rapid responses to the political stimuli in brain areas related to the processing and responses to affect (Homan, Hamdan, et al., 2023; Morris et al., 2003).

**Figure 2.**
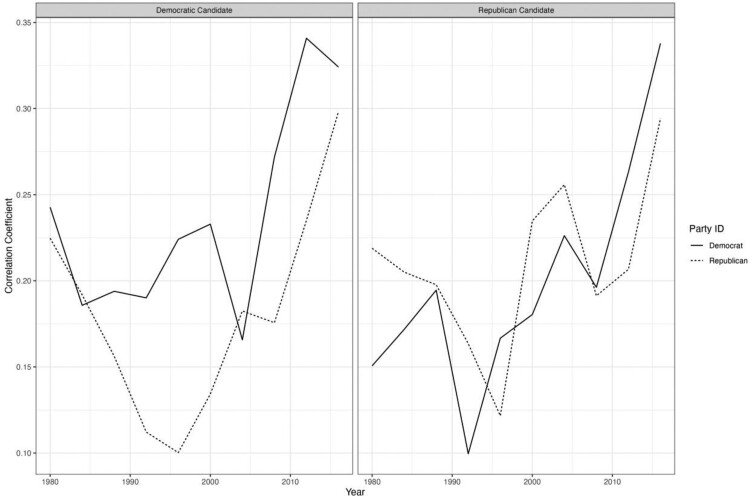
Mean correlation between emotions (anger, hopeful, afraid, and proud) felt towards each party's candidate by party identity and over time.

However, the concept of affective polarisation is built on theories of partisanship as a social identity, and these theories relied on intergroup emotions theory (IET) in their conceptualisation (Huddy et al., 2015). IET suggests several testable implications: First, “people's responses as members of a group are not idiosyncratic but are shared with other group members – if you are thinking about yourself as American, you report feeling about the same amounts of anger, hope, fear, and pride, for example, as other individuals thinking about themselves as Americans” (Mackie et al., 2008, p. 1869). The American National Election Study asked respondents whether the Democratic and Republican candidates made them angry, hopeful, afraid, or proud. We split respondents by their self-reported identity and calculated the correlation between emotions for each candidate by year. The results are consistent with the expectations of the IET model and the hypothesis that America is increasingly affectively polarised. Both Democrats and Republicans are, over time, increasingly reporting feeling similar emotions to one another (Figure 2).

**Figure 3.**
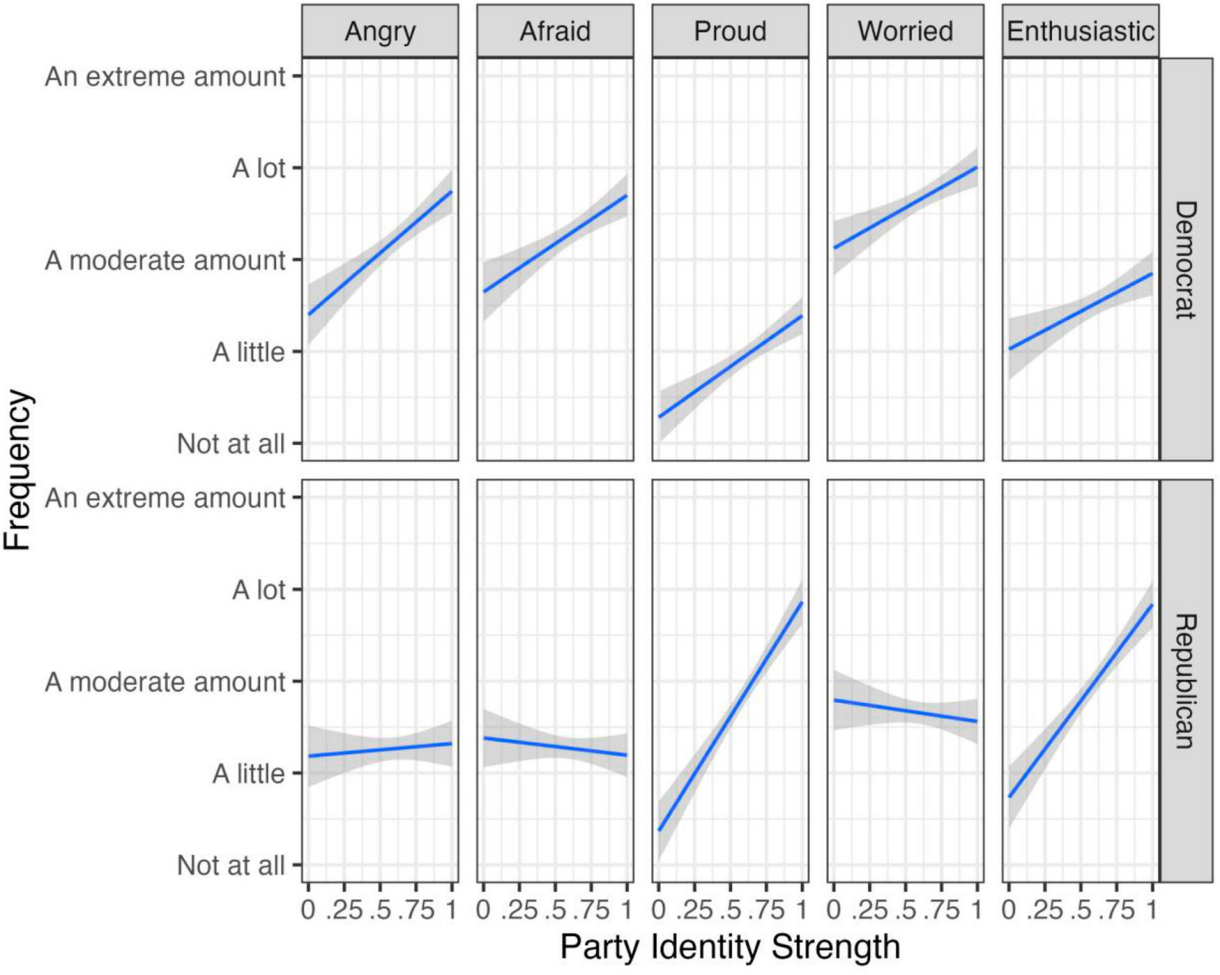
Frequency of each emotion felt thinking about the 2018 US congressional election by party identity strength and emotion.

Another empirical implication derived from IET is that “individuals for whom the group is central and important experience the emotions their group is feeling more intensely” (Mackie et al., 2008, p. 1869). Data from a nationally representative U.S. survey (*N* = 819) collected before the 2018 election (Miller, 2019) allows us to test this proposition. The researchers measured the centrality and importance of each person's party identification using a four-item measure developed by (Greene, 1999) (e.g. When talking about Democrats/Republicans, how often do you use “we” instead of “they”?), and then asked them “When you think about the outcome of the 2018 elections across the country, how much do you feel the following emotions” and were asked about anger, fear, pride, worry, and enthusiasm. Among Democrats, stronger partisans said they more frequently felt each emotion (both positive and negative emotions) than weaker partisans, although they were, on average, more negative than positive. Republicans tended not to experience negative emotions, but stronger partisans more frequently experienced positive emotions than weaker partisans (Figure 3).

Emotions are also related to partisanship outside the United States as well. Reporting the results from large surveys conducted in the UK and Norway, Berntzen et al. (2024) find strong positive correlations between disliking the political outgroup (e.g. the party a person feels furthest from) and reporting anger (UK: *r* = .52; Norway: *r* = .34), fear (UK: *r* = .33; Norway: *r* = .24) and disgust (UK: *r* = .52; Norway: *r* = .31). Berntzen et al. (2024) also asked respondents to report these emotions when thinking about the political ingroup (e.g. the party a person feels closest to). On average, the strength of the associations between outgroup liking and negative emotions is stronger than the strength of associations between ingroup liking and emotions – note that the authors did not formally test this, but we conclude this based on the size of the correlation coefficients reported in Berntzen et al. (2024). These findings align with work by Brandt and Vallabha (2022) that strengthening partisan identity (within individuals) is associated with increased affective polarisation in both the US and the Netherlands.

### Which emotion is politically important?

One reason affective polarisation has attracted so much attention is that hating the other side purportedly destabilises democracy as it reduces electoral accountability, leads to blind conformity, and a deterioration of democratic norms, including support for violence (Iyengar et al., 2019). For instance, Iyengar and Westwood (2015, p. 215) argue that “heightened [affective] polarisation has made it almost impossible for partisans to abandon their party's candidates, no matter their limitation”, and Iyengar et al. (2012, p. 428) writes, “partisan bias in perceptions of economic conditions means that voters will fail to credit opposing-party incumbents when the economy grows under their stewardship and fail to penalise in-party incumbents whose economic performance is suspect”.

However, the extant evidence on whether disliking the other side affects democratic norms is mixed. While affective polarisation is sometimes (and weakly) correlated with anti-democratic attitudes (Berntzen et al., 2024), studies that manipulate dislike of the other side in the lab do not find a direct relationship. For instance, Broockman et al. (2023) manipulate dislike of the other side through a modified dictator game, wherein they are led to believe they are playing another participant from the opposite party who is particularly generous. While the treatment increased how much respondents reported liking the outparty (and had a smaller effect on how much they reported liking the inparty), the treatment had no downstream effect on various anti-democratic attitudes. Similarly, in a “mega-study” that tested many different interventions to reduce affective polarisation, most interventions that changed feeling thermometers, e.g. increasing interparty contact or priming a common identity, did not affect support for undemocratic practices or support for partisan violence (Voelkel et al., 2022). This work suggests that the hypothesis that affective polarisation (and espically the hate towards the political out-group) leads to antidemocratic attitudes may not be correct.

One possible explanation is that the literature on affective polarisation has focused on the wrong emotion. In particular, the link between affect and anti-democratic attitudes may be due to fear, not loathing. As Braley and Lenz (2023) write:

The fear of attack by the other side often leads to a logic of preemptive strike, a theory most clearly laid out in international relations in terms of the “security dilemma.” If such dynamics are playing out within democratic politics … toxic polarisation and democratic backsliding could be characterized by a sort of “subversion dilemma” where increasing tensions between partisan groups could lead to preemptive strikes on the institutions of democracy.

The finding that beliefs directly affect anti-democratic attitudes but not necessarily (or at what is picked up by feeling thermometers) preclude anger, in particular, and other emotions, in general, from indirectly affecting anti-democratic attitudes.

Affect may bias both the information people encounter and how they process information, leading them to misperceptions of the other side. Emotions affect political information seeking (Albertson & Gadarian, 2015). Anxiety, for instance, leads people to broaden their information search (Valentino et al., 2009) and to seek information that is the cause of the threat (Gadarian & Albertson, 2014). For instance, anxiety about COVID-19 leads people to consult information they would typically not consult (Mehlhaff et al., 2023). Additionally, disgust shifts attention away from the topic (Clifford & Jerit, 2018; Nabi, 1998). Disgust might lead people to turn their attention away from politics altogether.

Emotions may bias political information processing. In particular, partisan animosity may distort which information people consume and how they process information. For instance, the term affective polarisation is often used interchangeably with social distance. A person who dislikes the other side will not only avoid interacting with out-party members (Lelkes & Westwood, 2017), but they may also avoid information associated with that side or consume information associated with their side. For instance, an affectively polarised Republican may avoid MSNBC and primarily consume Fox News, or, on social media, they may selectively follow members of their party (or unfollow members of the other party) (Kaiser et al., 2022; Mosleh et al., 2023).

While several studies have argued that selective exposure to information increases affective polarisation, these claims are built mainly on correlational evidence between information consumption and partisan disdain. It seems possible that partisan disdain also leads to selective exposure. If affect conditions exposure to problematic information and problematic information has a direct effect on anti-democratic attitudes, then partisan disdain may indirectly impact anti-democratic attitudes.

Even if a person does not purposefully consume much political information–one paper finds that less than 1.6 percent of online web browsing is to political news content (Wojcieszak et al., 2024)–people can still learn about politics from everyday discussion, both in person (Druckman et al., 2018) and online (Settle, 2018). Hence, if people's information environment is skewed partly because of partisan disdain, people will have a skewed perception of reality. This problem is likely exacerbated by a media environment that rewards angry and sometimes untruthful information about the other side. For instance, a person who, because they dislike Democrats, only hears information from Fox News may reasonably, believe that Donald Trump was the perfect president.

Similarly, not only are those that are vocal on social media more likely to be partisan extremists (Bor & Petersen, 2022; Kim et al., 2021), but those posts that are more partisan and more vitriolic are more likely to receive social media engagement (reposts, likes, comments) (Rathje et al., 2021). An eye-tracking study by Kohout et al. (2023) showed that negative, and in particular, angry comments receive the most visual attention. As posts that receive more engagement are also more likely to get boosted by social media algorithms, many people receive a distorted picture of reality that paints the other side as extreme or a threat to democracy, potentially triggering the subversion dilemma (Braley et al., 2023).

Even if people have a balanced information diet or exist in heterogeneous networks, disdain may bias how they process that information. In a Bayesian model of information processing, people's posterior attitudes are a function of their prior attitude, the new information, and the weight they put on it. That weight is partly determined by how much they trust the source. One common operationalisation of affective polarisation is trust (Iyengar & Westwood, 2015). Those who dislike the other side trust their side more and the other side less. Hence, if two Democrats receive identical information about Republican extremity from a Democratic source, the more disdainful partisan will put more weight on the new information than the less disdainful partisan.

Anger may be one emotion biasing information processing (Moons & Mackie, 2007). Anger increases a person's reliance on potentially polarising heuristics (Huddy & Khatib, 2007). A less mindful partisan may glom onto certain information because it comes from an ingroup member, which can potentially increase disdain and anti-democratic attitudes. At the same time, anger facilitates directional motivated reasoning. For instance, Young et al. (2011) finds that anger leads to seeking disconfirming information – which the angry citizen could use to counterargue. People become less interested in learning more about candidates with whom they disagree (Redlawsk et al., 2007). Instead, people seek out information that supports their priors when angry (MacKuen et al., 2010) and evaluate in a biased way. As Erisen (2020, p. 11) summarises, “anger causes people to both lean heavily on their prior dispositions and respond in a hostile manner toward people and ideas that undermine them”, which makes “the case for anger as a major predictor of politically biased assimilation … compelling”.

### Why so negative? The role of positive emotions

The affective polarisation literature has primarily focused on negative emotions. This is partly driven by a rise in America of out-party antipathy (as measured by feeling thermometers) but relative stability in-party warmth. However, researchers should (and increasingly are) focusing on the positive emotions as well (Webster & Albertson, 2022). Positive emotions play a central role in one influential conceptualisation of partisanship as an expressive social identity (Huddy et al., 2015). Partisans, particularly strong partisans, feel joy when their group's status is elevated. In a series of experiments Huddy et al. (2015) find that those with the strongest attachment to “their team” expressed the highest self-reported enthusiasm when they were informed that their party was doing well in the polls compared to those with weaker ties to their party. Furthermore, exposure to information about in-party politicians versus out-party politicians increases enthusiasm dramatically (Bakker, Schumacher, and Homan, 2020).

Although a social identity framework helps us understand the dynamics in feelings towards the parties, in one way, partisanship qua a social identity is unlike other social identities. Social psychology has long suggested that ingroup love is primary (Brewer, 1999). That is, people first develop affection for a group and are primarily driven by a desire to increase the status of that group rather than a desire to quash the status of another group. However, according to at least one measure, while ingroup affection has remained relatively stable over the past 40 years, negative feelings towards the out-party have increased dramatically (Abramowitz & Webster, 2018; Iyengar et al., 2012). In 2020, people felt as warm towards their party as they felt cool towards the outparty, and those who very weakly identified with a party (partisan leaners) tended to dislike the outparty more than they liked their party (Lee et al., 2022). Similarly, researchers have modified a prisoner's dilemma-type behavioural game to disentangle out-group enmity from in-group amity. While ingroup amity swamps outgroup enmity for other social identities (of a person) that have been tested (Weisel & Böhm, 2015), including German political identities, partisans in America engage in as much outgroup hostility as ingroup favouritism (Lee et al., 2022).

This is not to say that ingroup love is not the primary driver of affect and behaviour. Tajfel (1974, p. 66) argued that people “must first have acquired a sense of belonging to a group which is distinct from the one they hate, dislike or discriminate against”. Based on models wherein some behaviour is regressed, simultaneously, on out-party antipathy and in-party warmth, many papers have claimed that negative affect is more important than positive affect (e.g. Abramowitz & Webster, 2016; Rathje et al., 2021). However, if feelings towards the inparty drive feelings towards the out-party, these models include both the antecedent and the mediator (Lelkes, 2021a).

While the coefficient on negative feelings is often larger than the coefficient on positive feelings, the total effect of positive feelings is larger. When people are explicitly asked if they identify with a party because they like their side (positive partisans) or oppose the other side (negative partisans), most people say they are “positive” rather than “negative” partisans (Lee et al., 2022). The one exception is partisan leaners–people who only identify with a party when pushed by the survey researcher–tend to be negative partisans.

Partisans also experience positive emotions such as joy, or at least joy in the suffering of outpartisans. Schadenfreude can be a group emotion, wherein individuals experience joy when misfortunate befalls a rival group or rival group member (Nai & Otto, 2021). Recent literature has introduced the concept of “partisan schadenfreude” (Kalmoe & Mason, 2022; Webster et al., 2022): the idea that strong partisans experience joy and pleasure in seeing partisans from the other party (team) suffer. Furthermore, a non-trivial segment of the U.S. population would support a candidate who promises to inflict pain on their political rivals (Webster et al., 2022).

Another ostensibly positive emotional process–empathy–has also been linked to political behaviour. Simas et al. (2020) and Schumacher et al. (2023) find a positive association between levels of empathy (empathic concern) and levels of affective polarisation, and experimentally increasing empathy increases partisan bias. Interestingly, across the two studies, the effects for another subdimension of empathy perspective taking – are more mixed: while Simas et al. (2020) find no systematic effects for perspective taking on affective polarisation, Schumacher et al. (2023) show that increased perspective taking reduces affective polarisation.

These positive emotions, however, can have negative consequences. Strong partisans tend to become the most enthusiastic, and enthusiasm drives political engagement (e.g. Groenendyk & Banks, 2014). Because strong partisans tend also to be more politically extreme and politically active, they may vote for more extreme candidates in political primaries. Extreme political candidates then polarise the mass public (Banda & Cluverius, 2018). Hence, emotions not only have individual-level effects on political behaviour, but they also (potentially) have system-level effects as well. This interplay also suggests that a complex systems approach may be particularly suited to modelling and understanding how micro-processes interact with system-level dynamics (Butler, 2022; Levin et al., 2021).

Let us illustrate a few studies that relied upon a complex systems approach. Kawakatsu et al. (2021), for instance, argue that the strength of partisanship conditions the extent to which people are willing to learn from the outparty. This biased transmission facilitates pairwise cooperation, while simultaneously increasing system-level polarisation. In another example, Goldenberg (2024, p. 3) uses a complex systems approach to uncover the “processes that contribute to an increase in the duration and intensity of collective emotion”. In this model, interactions involve the influence of one person's emotions on others, cognition encompasses various processes of perceiving and evaluating others’ emotions, and infrastructure refers to spaces enabling emotional interactions and cognitions, primarily focusing on venues like interpersonal conversations, small group meetings, online gatherings, and social media discussions where political emotions are shared. Adopting this framework would help us uncover the mechanisms underpinning affective polarisation and identify potential solutions. For instance, can we design spaces that are better at reducing polarisation and increasing conversation health (Carlson & Settle, 2022)?

### Conceptualising and measuring “affect”: beyond the feeling thermometer

Exploring affect with survey self-reports- the dominant way of measuring affective polarisation- is a little like looking for your lost keys under a street lamp. Political scientists search for affect where it is easiest to find (and measure). Still, the validity, importance, and generalizability of these self-reports as indicators of the broad concept of “affect” is an open question.

If we adopt a more holistic conception of emotion, the study of affective polarisation becomes far more nuanced. Emotions, according to Keltner and Gross (1999, p. 468), are “episodic, relatively short-term, biologically based patterns of perception, experience, physiology, action, and communication that occur in response to specific physical and social challenges and opportunities”. Following this definition, self-reported discrete emotions are just one part of the affective experience. Yet, there must be a much more comprehensive range of more unconscious or conscious responses that make up the “affect” in affective polarisation. If only because self-reports are quite limited in capturing the full complexity of affect (e.g, Dukes et al., 2021; Fontaine et al., 2022; Sauter & Russell, 2023; Scherer & Moors, 2019; Schiller et al., 2024).

One way to think about “affect” in polarisation is to build upon dimensions of core affect (Russell, 2003) and its more recent updates (Fontaine et al., 2022; Schiller et al., 2024). Affect, in this line of theorising, is conceptualised as having multiple dimensions: *valence*, which refers to the assessment of whether a state is positive or negative, and arousal, which pertains to the level of physiological activation of the autonomic nervous system. Aside from valence and arousal, there is an ongoing discussion about whether there are two additional (power and novelty) (e.g. Fontaine et al., 2022) or one (motivation) (Schiller et al., 2024) – an important discussion but not one we need to tackle in this article. Importantly, affective polarisation research has largely only considered the second dimension (arousal) and done so primarily using survey self-reports or behavioural outcomes. There is, however, a burgeoning literature on arousal and a more expansive view of valence in politics.

In the following section, we review some work in political science, psychology, and related disciplines suggesting polarisation affects outcomes beyond survey self-reports. Some of these studies explicitly frame their research towards (affective) polarisation, while others do this more indirectly. This is not a systematic literature review, but instead, we bring together a selection of studies that have broadly measured affect using neurobiological theories and methods – broadly conceived – such as heart rate, skin conductance, and fEMG. These studies have different designs and goals, but across the board, they investigate the affective responses to exposure to information that either supports or comes from one's political party versus information that either supports or comes from the out-party.

#### Politics and physiological arousal

Arousal captures the intensity of the affective responses. Skin conductance, generally regarded as an indicator of arousal, measures the activity of the autonomic nervous system, specifically the sympathetic nervous system (Dawson et al., 2017). As sweat secretion increases, the conductance of electricity improves, and the skin conductance levels increase. Increased skin conductance (and thereby arousal) have been reported in response to negative images (Lang et al., 1993). In the domain of politics, skin conductance increases were captured in response to negative and threatening news (Boyer, 2023; Dubèl et al., 2024; Soroka et al., 2019) and exposure to uncivil political debates (Mutz, 2007; Mutz & Reeves, 2005). At the same time, skin conductance goes up in response to positive stimuli such as exposure to a preferred football team (Potter & Keene, 2012).

A few studies have found a link between inparty versus outparty information and skin conductance levels as captured arousal, whereby arousal increases in response to outparty information compared to inparty information (Schumacher et al., 2022; Wang et al., 2014). Furthermore, “those who are more physiologically reactive to anticipating a political discussion are more likely to discuss politics with co-partisans” and that the “physiological experience of interacting with disagreeable others may be a factor influencing the choices people make in structuring their discussion networks” (Carlson et al., 2020, p. 183). This has implications for polarisation as Carlson et al. (2020, p. 165) explain: “aversion to psychological and physiological discomfort induced by political discussions could contribute to social polarisation”.

Not all findings align: Bakker, Schumacher, et al. (2021) do not find, among a Dutch sample, a consistent impact of exposure to disagreement on neither skin conductance responses nor heart rate. Several studies other studies fail to find statistically significant differences in skin conductance responses to images of in-party and out-party politicians and logos (Bakker, Schumacher, & Homan, 2020; Petersen et al., 2015). To summarise, it's unclear at this point that partisan information triggers arousal.

#### Politics and valence

Valence, often considered the first dimension of affect (Fontaine et al., 2022; Russell, 2003), can be captured via facial Electromyography (fEMG) (Larsen et al., 2008). fEMG measures minute, fast, bioelectric signals at the skin, which are caused by muscle contractions in a specific region (Blascovich et al., 2014). The *corrugator supercilii* – the muscle above the eyebrow – is associated with negative affect: increased corrugator activity has been recorded in response to negative images and words (Hietanen et al., 1998; Lang et al., 1993), as well as threatening and negative news (Boyer, 2023; Dubèl et al., 2024).

A few studies have focused on the *corrugator* activity in response to inparty and outparty information. Participants exposed to images of the in-party and out-party politicians show an overall strong positive facial response (increased zygomaticus and decreased corrugator activity) when observing in-party politicians and a strong negative facial response (both increased zygomaticus and corrugator activity) to out-party politicians (Homan, Schumacher, et al., 2023). Similarly, two studies found that exposure to political messages that go against someone's political predispositions evoke greater corrugator activity than messages they agree with (Bakker, Schumacher, et al., 2021; Goudarzi et al., 2020). Finally, participants exhibit strong *labii* responses – which have been associated with disgust – when shown images of politicians from the out-party versus the in-party (Bakker, Schumacher, & Homan, 2020)

Two other studies, however, fail to find any link between corrugator activity and exposure to opposing political messages (Schumacher et al., 2022; Wang et al., 2014). But there is reason for some caution interpreting these latter studies: One relied on a small sample (Schumacher et al., 2022), and the other used actual political campaign ads, which subjects may have already been exposed to (thereby dampening the effect) (Wang et al., 2014).

## Conclusions: a plea to emotion researchers

Affective polarisation, some have argued, threatens the stability of democracies across the globe. Hence, understanding and finding solutions to the problem is too important to leave to any one academic discipline. Affective polarisation research, largely the focus of political science, must be better conceptualised and measured to be better understood. Psychologists, in particular, have the theoretical expertise to meet this challenge. We conclude with a non-exhaustive set of questions that require the attention of psychologists.

### What is affective in affective polarisation?

Affect does not have a consistent or clear definition in the affective polarisation literature. Generally, research should engage in careful concept explication: we define the concept and then adopt operationalizations that best capture this definition. Affective polarisation research has, instead, found existing measures of public opinion and defined the concept based on those measures. This has led to a fair bit of conceptual ambiguity and potentially incorrect conclusions about the nature and effects of affective polarisation. We do not argue that we should jump right to more invasive measures. Before we hook people up to wires or put caps on their heads, it is important to decide on the definition of the concept. Adopting a broader framework for affect (e.g. Fontaine et al., 2022; Russell, 2003; Sauter & Russell, 2023; Schiller et al., 2024) is needed.

### Does politics have unique affective dimensions?

It may be the case that affect in the political domain is no different than affect outside the political domain. If so, politics may be a context to which existing frameworks of affect can readily be applied. However, there are some reasons to believe that affect functions differently in politics. For instance, there are weaker social desirability constraints in the domain of politics, and people are willing to openly express their disdain for the other side (Iyengar & Westwood, 2015).

Hence, affect should be elicited similarly but in different contexts. For example, Homan, Schumacher, et al. (2023) examines facial mimicry in response to pictures of regular people versus pictures of politicians. In response to pictures of regular people, participants copied the emotional expression of the person in the picture. However, in response to pictures of politicians, facial response was conditional on the politician's party. In response to an ingroup politician, participants showed a strong positive facial response (increased zygomaticus and decreased corrugator). In response to an outgroup politician, they show a strong negative facial response (increased corrugator and zygomatics). This study demonstrates that comparing affect in different domains may yield different results. However, the inparty/outparty findings may be replicated in any setting where a person is faced with an ingroup/outgroup member. For instance, a person may also exhibit a strong facial response when shown a picture of someone of a different race or who supports a rival sports team.

### What is the interplay between more conscious and unconscious instantiations of affective polarisation?

In politics, experiential measures of affect, such as self-reported emotions, tend to be only weakly correlated with unconscious measures of affect, such as physiological responses (Bakker, Schumacher, et al., 2021). In response, some have argued that we should favour one measure over the other (e.g. Osmundsen et al., 2022). In contrast, others argued that these measures capture different aspects of the affective experience (Bakker, Schumacher, et al., 2021; Bakker, Schumacher, Gothreau, et al., 2020).

**Figure 4.**
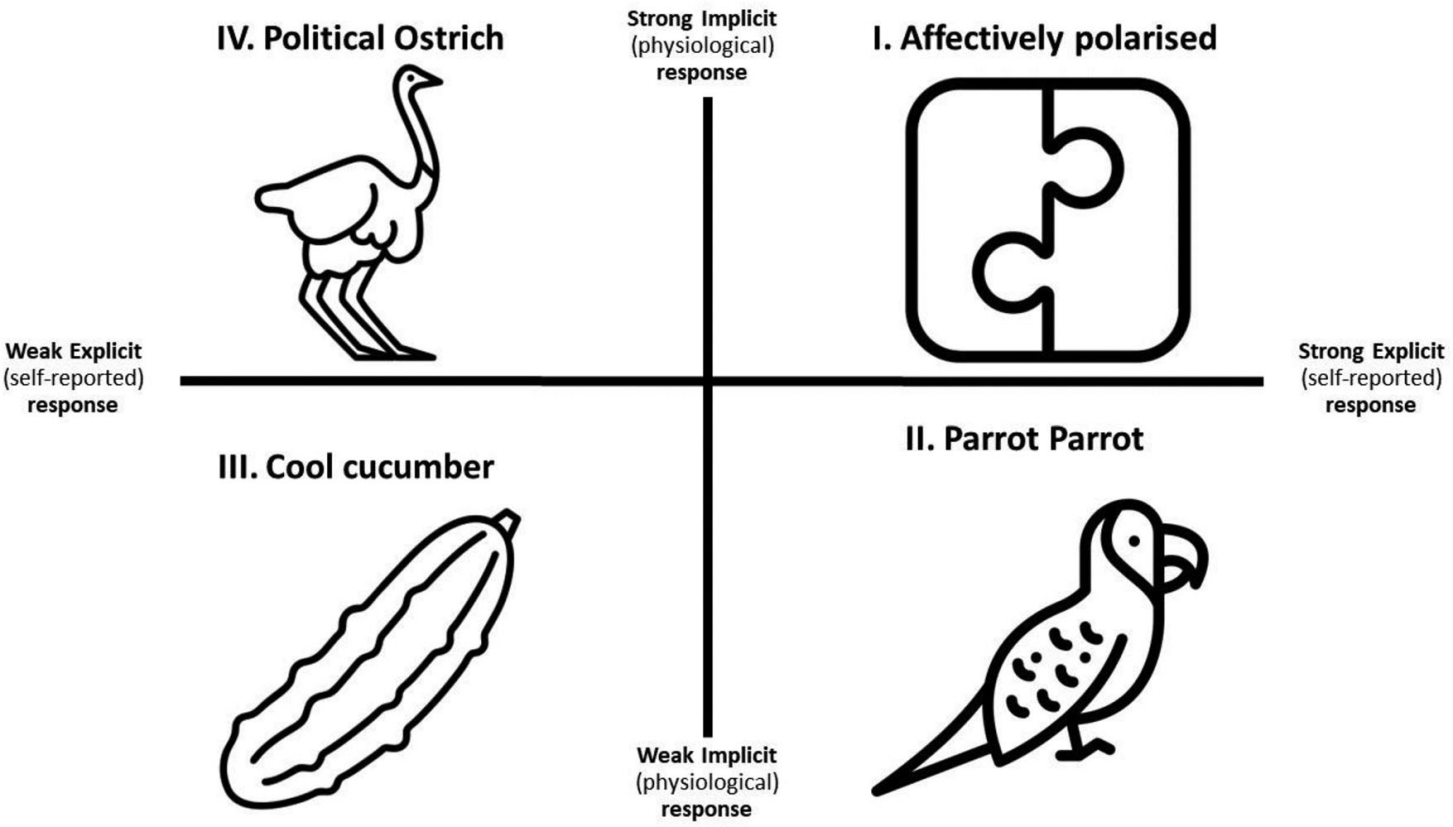
Model of concordance and discordance between explicit and implicit measures of affect as theorised by Arceneaux et al. (2024) and applied to affective polarisation.

In a recent paper, Arceneaux et al. (2024), argue that this incongruence has two important implications. First, there may be two routes to (de)polarisation, one that is unconscious and one that is more experiential. Bakker, Schumacher, et al. (2021) provide some evidence that this might be the case: self-reported anger and corrugator activity independently correlate with attitude change. Second, and building upon dual process theories (e.g. Evers et al., 2014), Arceneaux et al. (2024) argue there may be a variety of physiological and experiential patterns to political stimuli. We have adopted the model of Arceneaux et al. (2024) to the study of polarisation – see Figure 4 and discuss the model for valence (but the implications would be the same for arousal). The x-axis represents the self-reported, experiential, negative valence, and the y-axis represents the recorded physiological response, in the case of valence, which is corrugator activity.

This model allows us to disentangle the truly affectively polarised from those who respond in a purely expressive manner or mask their emotional responses to politics. Those in quadrant I are affectively polarised: they have strong physiological and experiential responses. For these people, affective polarisation might have the strongest positive (and negative) consequences in terms of (anti) democratic behaviour and non-political outcomes such as mental health (see below). Those in quadrant II express affect but don't have an affective response in terms of their physiological responses. They are “political parrots”. For them, expressing affect is part of being a loyal group member, and they are socialised into expressing affect as part of their group membership. Those in the bottom left corner (quadrant III), who may not experience any affective response to politics, are the least likely to adopt anti-democratic behaviour. However, they may also be detached from political life. People who have an unconscious response to politics but no experiential affect are in Quadrant IV. They are partisan ostriches who might have consciously, or unconsciously downregulated their affective response – Arceneaux et al. (2024) present initial empirical evidence for this model.

This model is a starting point for researchers to theorise about the role of affect in politics and the interplay between experiential and unconscious responses. Deeper theoretical development will allow researchers to identify unique mechanisms and targeted interventions for different groups of people.

### Does the role of affect in polarisation depend on the context?

While polarisation has been widely studied in the US, there is also a robust body of work on polarisation in other contexts (e.g. Bäck et al., 2023; Boxell et al., 2024; Garzia et al., 2023; Gidron et al., 2020; Hobolt et al., 2021; Hobolt et al., 2023; Janssen, 2024; Kekkonen & Ylä-Anttila, 2021; Wagner, 2021). The same is true for research on affect (e.g. Manokara et al., 2021; Sun et al., 2023). It would require large cross-context studies to investigate if and to what extent the interplay between social identities and affect have different dynamics depending on the contexts. On the one hand, one might expect that the dynamics are universal, yet culture and context could condition this relationship in unknown ways.

### Should interventions target emotion regulation strategies?

Most, if not all, of the research on the role of emotions in affective polarisation has focused on changing short-term emotional experiences in a particular context. However, these interventions, if effective at all (Voelkel et al., 2022), tend to have weak effects that dissipate quickly (Santoro & Broockman, 2022). A more fruitful approach may be to target emotion regulation strategies so that people have the tools to deal with polarising information. Emotion regulation strategies affect the emotions individuals experience, the timing of these emotions, and the manner in which they are felt and expressed. The emotion regulation literature suggests that when experiencing a negative emotion, people try to return to a pleasant emotional state. In the domain of politics, people may engage in cognitive reappraisal, wherein they reframe situations to change the emotional impact. They may down-regulate and lower the intensity of emotion. They may also attempt to distract themselves and direct attention away from the emotion (e.g. shifting attention from politics to other (non-)political topics that are not evoking emotions). Preliminary research in one context (US) on one explicit (self-reported), and arguably polarising, political actor (Trump) suggests that there is variation in the regulation strategies people use to deal with an emotion evoked by politics (Ford et al., 2023). Others have turned to mindfullness interventions and showed that brief mindfulness interventions can reducate affective polarisation (Simonsson et al., 2022).

An alternative approach involves exploring “collective” methods of regulating emotions, a concept described by Goldenberg (2023) as the process wherein a subset of a group engages in behaviour aimed at influencing the collective emotional response. The underlying notion behind collective emotion regulation processes is that as more group members become proficient in employing a specific emotion regulation technique, the resulting spill-over effect on the rest of the group intensifies. Pinus et al. (2023) have demonstrated that there is a significant relationship between the number of participants undergoing treatment and the reduction of negative emotions within a group, showing a substantial decrease in negativity as the proportion of treated individuals increases (Pinus et al. 2023, p. 2). Although the exploration of collective emotion regulation strategies is still in its early stages, this perspective could prove valuable in the study of affective polarisation. Affective polarisation is inherently group-based, and considering that it is impractical to individually treat an entire population, this approach offers a promising avenue for further research.

### What are the implications of affective polarisation, beyond politics?

Political scientists have primarily focused on affective polarisation's political and interpersonal consequences (Broockman et al., 2023). Politics also affects ostensibly non-political constructs such as sociodemographic identities (Egan, 2020), religiosity (Margolis, 2018), values (Connors, 2020), and personality (Bakker, Lelkes, et al., 2021). However, some evidence suggests that affective polarisation impacts two domains that are usually in the realm of psychology: mental health and adolescent development.

First, affective polarisation (or the perception thereof) is positively correlated with mental health outcomes such as increased levels of depression and anxiety symptoms (Fraser et al., 2022; Nayak et al., 2021; Smith, 2022). Tsakiris et al. (2021, p. 2) recently argued that polarisation, among other crises and stress factors in modern-day society, has:

a tangible effect on the political animals of the twenty-first century: they place them in a state of allostatic load. If one of the key functions of the brain is to serve the body by maintaining a healthy ‘body-budget’, then chronic or frequent stress depletes this budget, causing wear and tear to our regulatory systems, resulting in an allostatic load. In other words, we lose our ability to flexibly regulate our bodies. This results in compromised recovery and contributes to disease and poor health, emotional dysregulation and cognitive decline, and a vicious cycle that exacerbates the conditions that promoted allostatic load in the first place

While it is tempting to conclude that polarisation has negative *effects* on mental health, the arrow of causality could point the other way. Mental health may also impact affective polarisation and trigger a vicious and reinforcing cycle between mental health and polarisation –for a similar argument of the cyclical relationship between threat and politics, see Brandt and Bakker (2022) and Onraet et al. (2014).

Second, what does growing up in an affectively polarised society mean? Young children seem to be affectively polarised (Lay et al., 2023; Tyler & Iyengar, 2023). Children adopt the political attitudes and beliefs of their parents, and these attitudes and beliefs are increasingly hostile. Does it matter that kids are affectively polarised? At the individual level, the mental well-being of young people seems to be affected by politics (Smith, 2022). At the societal level, the future of democracy is at risk if young people enter the electorate already polarised. Democracy depends on citizen's ability to compromise (e.g. Wolak, 2020), engage in meaningful debate (e.g. Carlson & Settle, 2022; Hobolt et al., 2023), and objectively evaluate evidence (e.g. Druckman & McGrath, 2019). At the current moment, most citizens fail to meet this standard. While alarming, this also highlights opportunities for those interested in creating and testing school programs that help the youth fulfill their civic duty.

### A plea to psychologists

The study of affect in politics is too complex and important to leave to political scientists. In particular, we do not clearly understand the affective component of polarisation. Hence, the concept remains conceptually muddied and poorly measured. Effectively tackling the problem of affective polarisation requires insights from different disciplines, particularly those with clear expertise in emotion research. We hope this paper is read as an effort to introduce those interested in the topic to the extant literature (and its limitations) and a plea for psychologists to lend their expertise.

## Data Availability

The data and R-code to reproduce the results reported in Figures 1 and 2 can be found on our OSF page https://osf.io/y4jt7/. The data belonging to Figure 3 can be found here: 10.7910/DVN/XI1VKC.

## Note

1 This is not to say that policy and identity are orthogonal–instead, the policies and identities people adopt likely affect one another (Dias & Lelkes, 2022; Orr & Huber, 2020).
